# QRS Area Before and After Upgrading to Cardiac Resynchronization Therapy Is Not Sufficient to Predict Outcome

**DOI:** 10.3390/jcdd13070331

**Published:** 2026-07-14

**Authors:** Fenna Daniëls, Frederieke Eerenberg, Mariëlle Kloosterman, Antonius M. W. van Stipdonk, Cheyenne S. L. Chiu, Mathias Meine, Kevin Vernooy, Michiel Rienstra, Alexander H. Maass

**Affiliations:** 1 Department of Cardiology, University Medical Center Groningen, Hanzeplein 1, 9713 GZ Groningen, The Netherlands; f.daniels@umcg.nl (F.D.); m.kloosterman@umcg.nl (M.K.); m.rienstra@umcg.nl (M.R.); 2 Department of Cardiology, Cardiovascular Research Institute Maastricht (CARIM), Maastricht University Medical Centre, P. Debyelaan 25, 6229 HX Maastricht, The Netherlands; frederieke.eerenberg@mumc.nl (F.E.); twan.van.stipdonk@mumc.nl (A.M.W.v.S.); kevin.vernooy@mumc.nl (K.V.); 3 Department of Cardiology, University Medical Centre Utrecht, Heidelberglaan 100, 3584 CX Utrecht, The Netherlands; c.s.l.chiu-4@umcutrecht.nl (C.S.L.C.); m.meine@umcutrecht.nl (M.M.)

**Keywords:** paced QRS area, delta QRS area, upgrade to CRT, CRT-responder

## Abstract

QRS area reflects ventricular electrical dyssynchrony and is a stronger predictor of cardiac resynchronization therapy (CRT) outcome than QRS duration/morphology. Data on QRS area in patients upgraded from right ventricular pacing to CRT are scarce. We investigated whether QRS area and the difference with biventricular paced QRS area (ΔQRS) in CRT-upgraded patients are associated with clinical outcome and echocardiographic response. This retrospective multicenter study included patients upgraded to CRT. Median values were used as a cut-off for (Δ)QRS area. The primary composite outcome was all-cause mortality, heart transplantation or left ventricular assist device. Secondary outcomes were heart failure hospitalization (HFH) and ≥15% reduction in left ventricular end-systolic volume. A total of 303 patients were included (age 69 ± 11 years, 75% male, 50% ischemic cardiomyopathy). Median LVEF was 26% [25–34]; baseline QRS area was 132 µVs [97–162], which reduced to 90 µVs [61–122] with CRT (*p* < 0.001), with a ∆QRS area of 35 µVs [1–74]. The primary outcome occurred in 34.3% during 47 ± 29 months of follow-up without differences between a small or large baseline and ∆QRS area (*p* = 0.856; *p* = 0.968). HFH occurred in 9.2% and patients with large ∆QRS area had fewer HFHs (6.7% versus 15.4%, *p* = 0.012). Baseline QRS area of >132 µVs was not associated with HFH (*p* = 0.832), but was a determinant of better echocardiographic response (OR 2.23, 95% CI 1.10–4.50, *p* = 0.026). ∆QRS area of >35 µVs was not associated with echocardiographic response (*p* = 0.203). To conclude, (∆)QRS area was not associated with the primary outcome in this small cohort of CRT-upgraded patients. However, baseline QRS area of >132 µVs was associated with echocardiographic response and ∆QRS area of >35 µVs with fewer HFHs.

## 1. Introduction

Cardiac resynchronization therapy (CRT) has been shown to improve symptoms and reduce morbidity and mortality in patients with heart failure and left bundle branch block (LBBB) [1]. Delayed left ventricular lateral wall (LVLW) activation is considered the mechanism underlying left ventricular (LV) dysfunction, and may be improved through CRT. Unfortunately, not all patients with heart failure benefit from CRT. Recommendations in current guidelines are made to select patients with the highest likelihood of benefiting from CRT and are based on optimal medical therapy, left ventricular ejection fraction (LVEF), QRS duration and QRS morphology [2,3]. However, several studies showed that baseline QRS area is a stronger predictor for CRT outcome compared to baseline QRS duration or LBBB morphology [4,5,6], raising the question of whether QRS area should be used for selecting suitable CRT patients. Vectorcardiographically derived QRS area is a measure of LV electrical dyssynchrony and combines elements of QRS duration and morphology into one objective measurement [7]. A larger QRS area reflects (more) delayed LVLW activation. Although LBBB QRS morphology and broader QRS duration are known to be indicators of delayed LVLW activation, delayed LVLW activation can also be present in patients with non-LBBB and less pronounced QRS prolongation [7]. QRS area could therefore be helpful in identifying non-LBBB patients with an electrical substrate for CRT. In addition to baseline QRS area, a larger reduction in QRS area (∆QRS area) as a response to biventricular pacing is associated with significantly better outcomes in CRT patients [8,9,10,11], and may be useful to guide LV lead placement in CRT [12].

Previous studies regarding QRS area as a predictor for CRT outcome included only a few patients with upgrades from right ventricular (RV) pacing to CRT. In general, prediction models of response to CRT in upgrades are scarce [13]. Current data regarding upgrades are limited to observational controlled trials, registries and retrospective studies, usually compared with de novo CRT, and guideline recommendations refrain from paced QRS morphology or duration as indications for upgrade to CRT [3]. The BUDAPEST-CRT trial is the first randomized controlled trial comparing upgrade to CRT defibrillator (CRT-D) to implantable cardioverter defibrillator (ICD) with RV pacing, showing a substantial beneficial effect of CRT-D in a subset of patients [14]. However, this remains controversial, since the RAFT study showed no benefit in the subgroup of patients with RV pacing upgraded to CRT-D compared to ICD only [15]. The present study investigates whether the baseline paced QRS area in patients referred for an upgrade from RV pacing to CRT is associated with clinical outcome and echocardiographic response. Additionally, the association of the ∆QRS area with the outcome is studied.

## 2. Materials and Methods

In this study we used data derived from the MUG (Maastricht–Utrecht–Groningen) cohort [4]. Consecutive patients who received a CRT device between January 2001 and January 2015 in any of the three participating University Medical Centers in the Netherlands were retrospectively included. There were no formal inclusion or exclusion criteria on device implantation, patient selection or patient follow-up, which were done according to the then-prevailing guidelines [16]. Device optimization was up to the discretion of the patients’ physician. For inclusion in the database, all patients were required to have a baseline digital 12-lead electrocardiogram (ECG) and CRT had to be continued until end of follow-up in December 2015.

**Figure 1:**
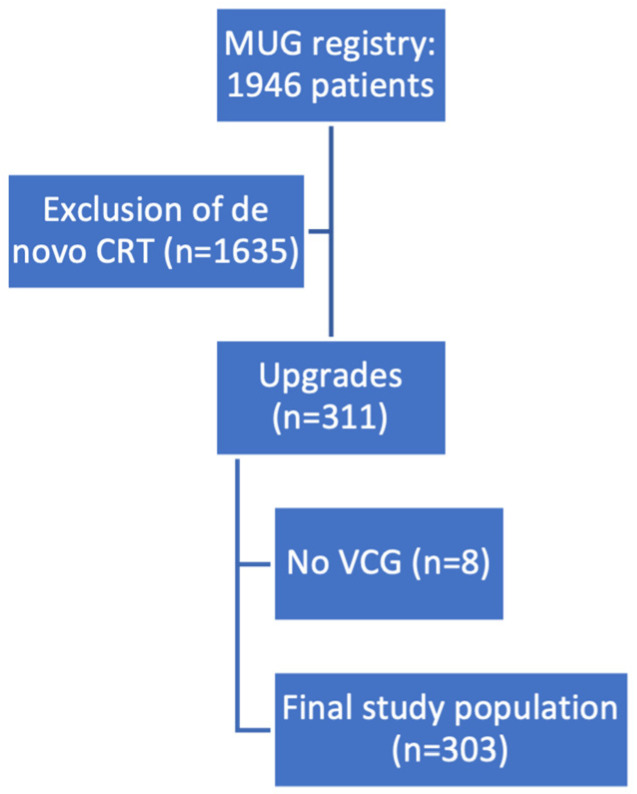
Patient selection. CRT = cardiac resynchronization therapy. VCG = vectorcardiogram.

In the MUG cohort, 1946 patients were included. For the present study we selected the 311 patients with right ventricular pacing who were upgraded to a CRT device. These patients had been excluded in the previously published studies from the MUG cohort. In 8 patients, vectorcardiographic analysis could not be performed due to frequent premature ventricular complexes, and they were therefore excluded from the database. The patient selection is shown in Figure 1.

Baseline data, such as heart failure cause and classification, medication, and comorbidity, were obtained from the local hospital electronic patient files. The etiology of heart failure was defined as ischemic when there was clear evidence of myocardial infarction, extensive coronary artery disease or coronary artery bypass grafting in a patient’s medical history. Device parameters were retrieved from specific device databases and LV lead location was evaluated by chest X-ray or fluoroscopic images. Post-implantation 12-lead ECGs with biventricular pacing were obtained and converted to vectorcardiograms. ∆QRS area and ∆QRS duration were calculated by subtracting biventricular paced values from their baseline value, where a positive value represents a reduction in QRS area and duration. Follow-up that was not performed at the implantation center was considered as missing data.

All data used were handled anonymously and this study was conducted in accordance with the principles of the Declaration of Helsinki. A non-WMO (Dutch law on Research Involving Human Subjects Act) declaration was received from the Medical Ethics Review Board of the University Medical Center Groningen. At the time of data collection of the MUG database, the Dutch Central Committee on Human-Related Research (CCMO [Centrale Commissie Mensgebonden Onderzoek]) allowed the use of anonymous data without prior approval of an institutional review board and without informed consent, provided that the data were acquired for routine patient care.

### 2.1. Electro- and Vectorcardiography

Recorded baseline RV paced and post-implantation biventricular paced 12-lead ECGs were stored digitally (MUSE version 8; GE Healthcare, Chicago, IL, USA) and automated ECG readings were used to evaluate ECG parameters. Paced QRS duration was measured from the QRS onset. For vectorcardiography, the original digital signals were extracted from the MUSE system and custom MATLAB R2010b software (MathWorks Inc., Natick, MA, USA) was used to convert the 12-lead ECG into three orthogonal vectorcardiography leads (X, Y and Z) using the Kors conversion matrix [17]. QRS area was calculated as the sum of the area under the QRS complex in the orthogonal vectorcardiography leads (QRS_area_ = (QRS_area,X_^2^ + QRS_area,Y_^2^ + QRS_area,Z_^2^)^1/2^). This method has been described previously [18].

### 2.2. Study Outcome

The primary outcome was a clinical outcome defined as the composite outcome of death, heart transplantation or left ventricular assist device (LVAD). Secondary outcomes were (1) (first) heart failure hospitalization (HFH), and (2) echocardiographic response, defined as a left ventricular end systolic volume (LVESV) reduction of ≥15% at six months follow-up. These outcomes were selected based on our earlier work demonstrating an association between QRS area and both clinical and echocardiographic response to CRT, in which patients who upgraded from RV pacing to CRT were excluded [4]. Additionally, data on LVEF were collected. All echocardiographic parameters were site-reported.

### 2.3. Statistical Analysis

Statistical analysis was performed using IBM SPSS version 28 (SPSS Inc., Chicago, IL, USA). Continuous, normally distributed, and discrete variables are presented as mean ± standard deviation and counts (percentages), respectively. Non-normally distributed values are presented as median [interquartile range Q1–Q3]. Cut-off values for a small and large QRS area were explored. A baseline non-paced QRS area of 109 µVs has previously been reported to be a determinant of outcome in CRT [4]. To ensure that the number of participants per group was equally distributed, the median value of our patient population was used as a cut-off value when this did not resemble 109 µVs. Since no optimal cut-off values of ∆QRS area have been reported, the median value was used. Kaplan–Meier survival analyses were used to evaluate the association between paced QRS area and ∆QRS area and the primary and secondary outcomes, with the log-rank test used to determine the difference in survival probabilities between groups with small and large paced (∆)QRS area. Cox and binary logistic regression analyses were used to assess univariate and multivariate-adjusted associations of QRS area with the primary and secondary outcomes. Hazard ratio (HR) and odds ratio (OR) are reported. The adjusting covariates were those known to be associated with primary and secondary outcomes of CRT in the literature. A two-sided *p*-value of <0.05 is considered statistically significant.

## 3. Results

A total of 311 patients were selected from the MUG registry. In 303 patients, the vectorcardiogram was available, representing the final study population.

### 3.1. Baseline Characteristics

The mean age was 69 ± 11 years; 75% of patients were men, 50% had ischemic heart disease, and 86% received CRT-D. The median ejection fraction was 26% [25–34] and most patients were in New York Heart Association (NYHA) class II (34%) or III (56%). A total of 70.4% of patients used a B-blocker in combination with an angiotensin-converting enzyme (ACE) inhibitor or angiotensin receptor blocker. Mineralocorticoid receptor antagonist use was unknown in 104 patients, and 44% of the remaining patients received a mineralocorticoid receptor antagonist. RV paced QRS duration was 188 ± 27 milliseconds (ms) and mean baseline QRS area was 137 ± 53 µVs. Median baseline QRS area was 132 µVs [97–162]. Baseline patient characteristics are shown in Table 1. The mean percentage of RV pacing, years of RV pacing, and indication for previous RV pacing were unknown.

### 3.2. Biventricular Paced Vectorcardiography

Biventricular paced 12-lead ECGs were available in 302 patients, of which 296 could be converted to a vectorcardiogram. Median biventricular-paced QRS area was 90 µVs [61–122]. Median biventricular paced QRS duration was 154 ms [136–173]. Median ∆QRS area was 35 µVs [1–74], which was a significant reduction compared to baseline (*p* < 0.001). Mean ∆QRS duration was 47 ± 2 ms, also significantly reduced (*p* < 0.001).

### 3.3. Clinical Outcome

The primary composite outcome of death, LVAD or heart transplantation occurred in 34.3% (*n* = 104) of patients during 47 ± 29 months of follow-up. There was no significant difference between a small or large QRS area (cut-off value of 132 µVs) and the occurrence of the primary outcome (*p* = 0.856). There was no significant difference between a small or large ∆QRS area and the occurrence of the primary outcome (*p* = 0.968).

**Figure 2:**
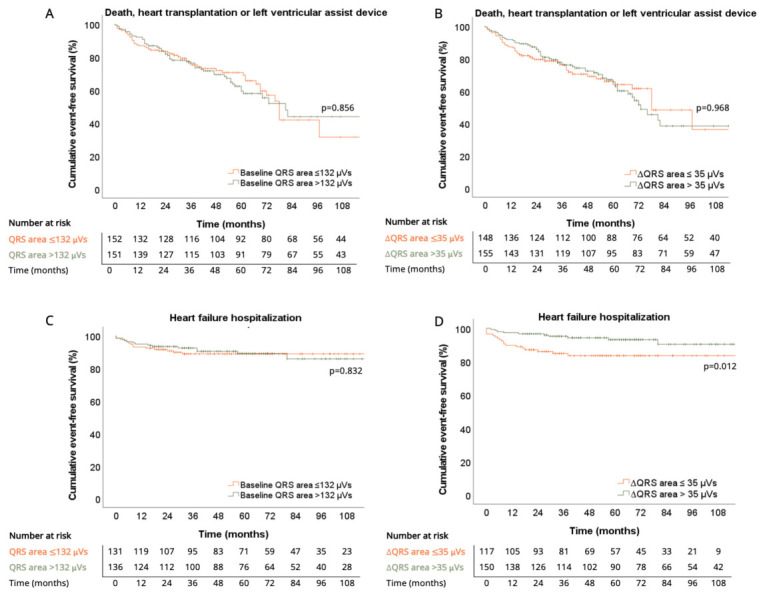
Kaplan–Meier. (**A**) Primary outcome of death, heart transplantation or left ventricular assist device for two groups with small and large baseline QRS area. (**B**) Primary outcome of death, heart transplantation or left ventricular assist device for two groups with small and large ∆QRS area. (**C**) Secondary outcome of heart failure hospitalization for two groups with small and large baseline QRS area. (**D**) Secondary outcome of heart failure hospitalization for two groups with small and large ∆QRS area.

Heart failure hospitalization occurred in 10.5% of the 267 patients with available follow-up during 43 ± 28 months and there was no difference in HFHs between a small or large baseline QRS area (*p* = 0.832). Patients with a large ∆QRS area showed significantly fewer HFHs compared to participants with a small ∆QRS area (*p* = 0.012), with, respectively, 6.7% and 15.4% of participants. Outcomes are shown in Table 2 and also illustrated in Figure 2.

### 3.4. Echocardiographic Outcomes

**Figure 3:**
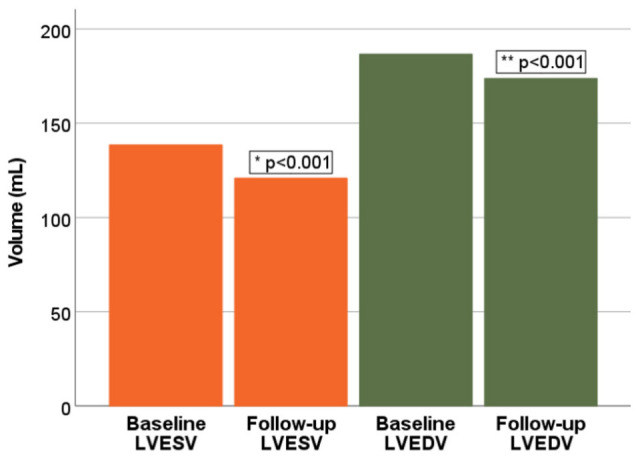
Echocardiographic data. LVESV, left ventricular end systolic volume. LVEDV, left ventricular end diastolic volume.

Echocardiographic follow-up was performed at 7 ± 6 months. Mean LVESV at follow-up was 121 ± 65 mL, with 78 patients (51% of available paired data) showing a reduction of 15% or more in LVESV, considered as echocardiographic responders. Mean absolute reduction in LVESV at follow-up was 18 ± 48 mL. Mean LVEF at follow-up was 33 ± 12% with 50 patients (31% of available paired data) showing >10% improvement in LVEF (Table 2, Figure 3). Among all baseline characteristics and clinical outcomes, no significant differences were observed between patients with and without available echocardiographic follow-up, except for baseline QRS duration and QRS area. Both measures were significantly larger in patients with available echocardiographic follow-up (*p* < 0.001 and *p* = 0.012, respectively). In contrast, changes in QRS duration and QRS area did not differ significantly between the two groups (*p* = 0.057 and *p* = 0.183, respectively).

### 3.5. Univariate and Multivariate Regression Analysis

Univariate associated covariates for the primary outcome were identified as age, body mass index (BMI), year of CRT device implantation, presence of ischemic cardiomyopathy and LVEF. Multivariate regression analysis showed that BMI per point (HR 0.90, 95% confidence interval (CI) 0.83–0.97, *p* = 0.004) and baseline LVEF per percent (HR 0.97, 95% CI 0.94–0.99, *p* = 0.032) were determinants of death, LVAD or heart transplantation. QRS area of >132 µVs and ∆QRS area of >35 µVs were not determinants of the primary outcome, as shown in Table 3. QRS area of >132 µVs was a residual confounder of echocardiographic response in LVESV reduction (OR 2.23, 95% CI 1.10–4.50, *p* = 0.026), together with hypertension (OR 2.52, 95% CI 1.21–5.25, *p* = 0.014) and diabetes mellitus (OR 0.30, 95% CI 0.11–0.83, *p* = 0.021), as shown in Table 4.

## 4. Discussion

In this study, baseline QRS area in patients with RV pacing and ∆QRS area was not associated with the primary outcome of the composite of death, LVAD or heart transplantation, but ∆QRS area of >35 µVs was associated with fewer heart failure hospitalizations (6.7% vs. 15.4%). On the other hand, a baseline QRS area of >132 µVs was a determinant of echocardiographic response with an odds ratio of 2.23. This is in line with the previous findings of Maass et al. in a larger population of CRT patients, also including de novo CRT. The CAVIAR score model, identifying predictors of response to CRT (defined as ≥15% LVESV reduction), showed that the QRS area was a predictor of response from a QRS area of >120 µVs, with increasing contribution as QRS area increased [6].

In addition to echocardiographic response, and in comparison to our study, other studies showed an association between baseline QRS area and clinical outcomes. Van Stipdonk et al. compared QRS area, QRS duration and morphology in their association with clinical and echocardiographic outcomes in 1492 de novo CRT patients. The baseline QRS area provided a better association with both clinical and echocardiographic outcomes compared with the combination of QRS duration and morphology. The median value of QRS area of 109 µVs was defined as the optimal cut-off value. Subgroup analyses showed that QRS area could especially improve patient selection in patients without typical LBBB and with QRS duration above 150 ms [4].

Three other studies included a small percentage of upgrade procedures [9,10,19]. In a retrospective study with 445 patients, of which 16% of the patients showed RV pacing at baseline, baseline paced QRS area (per 10 µVs) was a predictor of the occurrence of the primary endpoint of heart failure hospitalization or death (HR 0.46; [0.24–0.88], *p* = 0.019), but paced QRS area reduction was not (HR, 0.95; [0.82–1.1]; *p* = 0.47), which is similar to our findings [10]. Okafor et al. showed a pre-implantation QRS area of 102 µVs or a higher predicted cardiac mortality independent of QRS duration and morphology, with a hazard ratio of 0.36. Although this analysis included 13% of patients with RV pacing, no subanalyses were performed [9]. Friedman et al. studied whether outcomes of CRT could be predicted by the change in QRS area. Of the 527 participants, 91 patients had a RV paced baseline QRS morphology and the primary outcome was similar to our study. Different from the present study, ∆QRS area was divided into patient quartiles and progressive reductions in QRS area were associated with a lower rate of primary outcomes. However, baseline LBBB demonstrated predictive value and RV pacing at baseline did not [19].

The aforementioned association of a larger baseline QRS area with clinical outcome that was found in other studies in de novo CRT patients can be explained. First of all, it has been shown that QRS area is smaller in ischemic cardiomyopathy compared to non-ischemic cardiomyopathy, probably explained by the significant non-active scar that is present in ischemic hearts [20,21]. Moreover, women tend to have a larger QRS area compared to men [22], and QRS area is larger in patients with LBBB compared with other conduction abnormalities [4].

However, our study on QRS area is the first to only include patients upgraded to CRT, which could be one of the reasons why our findings do not correlate with the previously mentioned studies. RV paced QRS area does not account for the underlying intrinsic conduction or dyssynchrony, and therefore might not be a good predictor of outcomes. Moreover, patients upgraded to CRT are a specific population and have worse clinical outcomes compared with patients after de novo implantation. In a non-randomized multicenter observational study including 522 patients referred for CRT, with 177 upgrades, the upgrade procedures were associated with a lower response rate compared to de novo procedures (57% vs. 69%, respectively; *p* = 0.008). Moreover, survival at 37 months follow-up was lower in the upgrade group, with a hazard ratio of 1.65, with this effect remaining after adjusting for baseline variables [23]. The worse outcomes for upgrade procedures could be due to a greater surgical risk, but CRT might also have been initiated too late, including patients with advanced disease process and hence less chances for CRT to modify the risk. Also, the RV pacing may be an ‘innocent bystander’ or exacerbating factor instead of the cause of the heart failure, and hence resynchronization may only prevent possible future deterioration due to pacing-induced dyssynchrony, rather than immediately improve clinical and echocardiographic outcomes. Rath et al. studied predictors of response to CRT in patients with chronic RV pacing and included RV paced QRS duration and QRS reduction. Only the presence of non-ischemic cardiomyopathy and the use of ACE inhibitors were predictors of response; QRS duration during RV pacing, QRS reduction and percentage of RV pacing pre-implantation were not. However, response was defined as an improvement in NYHA class of at least 0.5 within 3 months or no heart failure hospitalization [13]. Additionally, Jastrzebski et al. studied the effect of QRS duration reduction on a combined endpoint of all-cause mortality and heart failure hospitalization in 552 CRT patients. A total of 18% of patients received RV pacing at baseline. Although a reduction in QRS duration by CRT was associated with a reduction in all-cause mortality and heart failure hospitalization, subgroup analyses showed that this was only significant in patients with baseline LBBB and not with RV pacing [24]. This implies again that the RV paced ECG does not reflect the underlying intrinsic conduction and therefore might not be a suitable parameter to predict CRT response. However, it is a subgroup analysis, and it might not be fair to compare changes in patients with RV pacing directly to patients with LBBB, as LBBB has been widely identified as a predictor of response to CRT.

Of note, a high percentage of 45.2% of patients in our cohort had atrial fibrillation (AF). Although not specified in our cohort as to whether this was paroxysmal or permanent AF, AF is known to reduce biventricular pacing percentages and induce atrioventricular dyssynchrony, thus reducing response to CRT [25]. Additionally, patients with AF and heart failure have higher mortality rates compared with heart failure patients without AF [26]. In our study cohort, AF was not significantly associated with either the primary clinical or echocardiographic outcome in univariable analysis. However, the study may have been underpowered to detect subgroup-specific differences, and data on biventricular pacing percentages were unavailable.

Although not all patients with RV pacing benefit from upgrade to CRT, the BUDAPEST-CRT trial is the first prospective randomized controlled trial to investigate outcomes after CRT upgrade and is in favor of CRT. A total of 360 patients with RV pacing for at least 6 months and at least 20% pacing burden, without native LBBB, and with paced QRS of >150 ms and LVEF of ≤35%, were randomized in a 3:2 ratio to CRT-D (*n* = 215) or ICD alone (*n* = 145). Over a median of 12.4 months, the primary outcome of heart failure hospitalization, all-cause mortality or <15% reduction in LVESV occurred in 32.4% of patients in the CRT-D arm and 78.9% in the ICD arm (OR 0.11 [0.06–0.19), *p* < 0.001). The incidence of procedure- or device-related complications was similar between the two arms [14].

**Figure 4:**
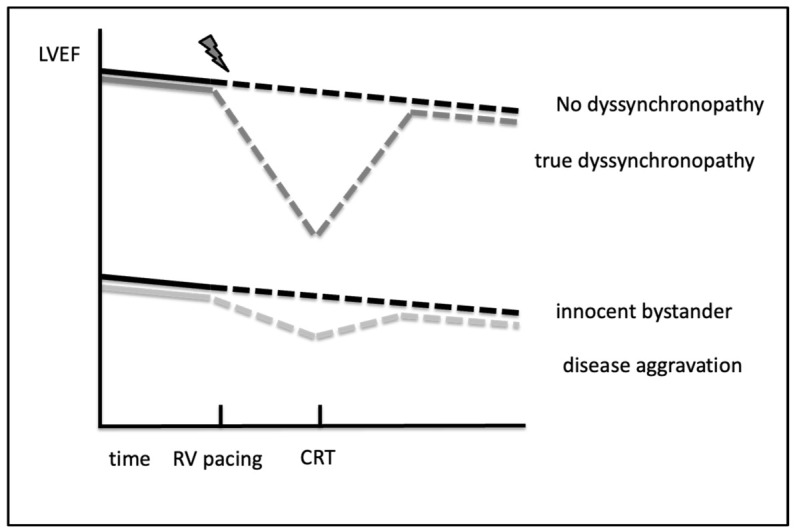
Dyssynchrony in patients upgraded to cardiac resynchronization therapy (CRT). This figure describes two scenarios in patients with right ventricular (RV) pacing upgraded to CRT with preserved or reduced left ventricular ejection fraction (LVEF) at baseline. The black line represents the natural course of disease, whereas the gray line represents the change in LVEF with RV pacing and CRT respectively. The upper two lines show the patients where RV pacing causes true dyssynchronopathy with LVEF deteriorating after RV pacing and improving to normal after CRT (typical super-responder). The lower two lines represent the patients where RV pacing is an innocent bystander and where the cardiomyopathy is caused by disease aggravation. RV pacing causes a slight deterioration in LVEF which is corrected after CRT is initiated.

Our study shows that patients with RV pacing have a relatively large QRS area, with a median value of 132 µVs, compared to other studies with non-LBBB, LBBB and biventricular pacing. For example, reported values of median QRS area are 77 µVs (non-LBBB), ranging between 101 and 145 µVs (LBBB) and ranging between 74 and 90 µVs (biventricular pacing) [5,10,27]. This is also shown in Table 5. However, this measurement of dyssynchrony was not the predictor of clinical outcomes, thus raising the question of whether it is the dyssynchrony itself that causes the deterioration of left ventricular function. Again, RV pacing could also be an ‘innocent bystander’, with deterioration of left ventricular function caused by progression of the underlying disease, as shown in Figure 4. Although the BUDAPEST-CRT trial did show the beneficial effect of CRT, further analyses should be awaited on subgroups of response, thereby identifying a specific patient population in which cardiac function is decreased by dyssynchrony by RV pacing, but is also reversible by upgrading to CRT. Additionally, if electrocardiographic data are available, it would be interesting to analyze the association of baseline QRS area with clinical and echocardiographic endpoints in the BUDAPEST-CRT population.

### Limitations

Several limitations should be acknowledged. First, information on the percentage of RV pacing was not available. Unfortunately, this is one of the parameters that has not been transferred to electronic patient files and could therefore not be retrieved in the majority of patients. Since the percentage of RV pacing increases the chance of pacing-induced cardiomyopathy, this could have been helpful in differentiating between pacing-induced left ventricular dysfunction or disease progression. Moreover, in patients with a lower percentage of RV pacing and therefore more often intrinsic conduction, the effect of CRT could have been different. Additionally, data on the percentage of biventricular pacing were unavailable. As a higher percentage of biventricular pacing is a known predictor of CRT response, this may have influenced the observed outcomes [28,29]. In addition, NTproBNP was not systematically available in this study. Baseline NT-proBNP levels were available for only a subset of patients, whereas serial measurements were not routinely collected. NTproBNP is associated with clinical outcome and baseline NTproBNP levels may also predict echocardiographic response to CRT [30,31]. Therefore, it would have been of interest to investigate the role of this biomarker in our study population.

The present study included patients who underwent CRT implantation between 2001 and 2015, a period during which CRT indications, implantation techniques, device programming and heart failure management evolved. Although the year of implantation of the CRT device was not independently associated with the outcomes in the multivariate analysis, extrapolation of our findings to contemporary patients should be done carefully. In addition, conduction system pacing is rapidly gaining ground as an alternative approach to deliver CRT, although biventricular CRT is still the established standard of care [32]. Furthermore, guideline-directed medical therapy for heart failure has improved over time [33]. In our cohort, the use of heart failure medical therapy was limited. Mineralocorticoid receptor antagonist use was unknown in 104 patients, and only 44% of the remaining patients received a mineralocorticoid receptor antagonist. A total of 70.4% of patients used a B-blocker in combination with an ACE inhibitor or angiotensin receptor blocker, whereas sodium–glucose cotransporter 2 inhibitors had not yet been introduced for the treatment of heart failure.

The relatively small sample size precluded subgroup analyses according to small versus large baseline QRS area or ∆QRS area and may have limited statistical power for the primary clinical endpoint. Although echocardiographic response was observed, a larger cohort and longer follow-up duration might have enabled the detection of differences in the clinical primary endpoint.

Finally, echocardiographic (follow-up) data were available in fewer than half of the patients, requiring cautiousness in drawing conclusions on echocardiographic response outcomes. However, baseline characteristics and clinical outcomes were largely comparable between patients with and without available paired echocardiographic data. In addition, other echocardiographic parameters of dyssynchrony would have been valuable. For example, apical rocking was significantly associated with the amount of reverse ventricular remodeling after CRT [6]. Unfortunately, this was not systematically available.

## 5. Conclusions

Baseline QRS area and ∆QRS area in this small cohort of patients with RV pacing referred for upgrade to CRT were not associated with the primary outcome of the composite of death, LVAD, or heart transplantation. However, a large baseline QRS area of >132 µVs was a determinant of echocardiographic response and a large ∆QRS area of >35 µVs was associated with fewer heart failure hospitalizations. Further and larger studies are required to establish whether there is a place for baseline paced QRS area and paced QRS area reduction as a predictor of response to CRT, possibly by exploring other cut-off values.

## Figures and Tables

**Table 1:** Patient characteristics.

Patient Characteristics	Total Population (*n* = 303)
Male sex	227 (74.9%)
Age, years	69 ± 11
BMI, kg/m^2^	27 ± 4
CRT-D	261 (86.1%)
*LV lead position* Anterior Anterolateral Lateral Posterolateral Posterior	*12 missing* 5 (1.7%) 33 (10.9%) 94 (31.0%) 142 (46.9%) 17 (5.6%)
Ischemic cardiomyopathy	150 (49.5%)
Diabetes mellitus	60 (19.8%)
Hypertension	127 (41.9%)
*NYHA class* I II III IV	*12 missing* 7 (2.3%) 102 (33.6%) 170 (56.1%) 12 (3.9%)
LVEF, % (103 missing)	26 [25–34]
LVESV, mL (117 missing)	137 ± 64
LVEDV, mL (116 missing)	186 ± 72
*Medication* B-blocker ACE inhibitor ARB B-blocker + ACE inhibitor/ARB Diuretics Digoxin Statin Anti-arrhythmics	*4 missing* 244 (80.5%) 187 (61.7%) 82 (27.1%) 214 (70.4%) 231 (76.2%) 37 (12.2%) 175 (57.8%) 58 (19.1%)
Atrial fibrillation	137 (45.2%)
QRS duration, ms	188 ± 27
QRS area, µVs	137 ± 53

BMI, body mass index. CRT-D, cardiac resynchronization therapy defibrillator. LV, left ventricular. NYHA, New York Heart Association. LVEF, left ventricular ejection fraction. LVESV, left ventricular end systolic volume. LVEDV, left ventricular end diastolic volume. ACE, angiotension-converting enzyme. ARB, angiotensin receptor blocker.

**Table 2:** Follow-up.

Follow-Up	Total Population (*n* = 303)
LVEF @ 6 months, % >10% improvement in LVEF	33 ± 12 (*n* = 182) 50 (31%) (*n* = 160)
LVESV @ 6 months, mL >15% reduction in ESV >Absolute reduction in ESV	121 ± 65 (*n* = 178) 78 (51%) (*n* = 152) 18 ± 48 (*n* = 152)
LVEDV @ 6 months, mL	175 ± 73 (*n* = 177)
*NYHA class* I II III IV	*(n = 232)* 32 (13.8%) 137 (59.1%) 61 (26.3%) 2 (0.9%)
LVAD, heart transplantation or death	104 (34.3%)
Heart failure hospitalization	28 (10.5%) (*n* = 267)
Biventricular paced QRS area, µVs	90 [61–122] (*n* = 296)
Biventricular paced QRS duration, ms	154 [136–173] (*n* = 296)
∆QRS area, µVs	35 [1–74] (*n* = 296)
∆QRS duration, ms	47 ± 2 (*n* = 296)

LVEF, left ventricular ejection fraction. LVESV, left ventricular end systolic volume. LVEDV, left ventricular end diastolic volume. NYHA, New York Heart Association. LVAD, left ventricular assist device. In case of missing data, the number of participants in which data are available is described. Percentages are percentages in the population with available data.

**Table 3:** Univariate and multivariate Cox regression analyses for primary outcomes (LVAD/Htx/death).

	HR	95% CI	*p*-Value	HR	95% CI	*p*-Value
	Univariate Analysis			Multivariate Analysis		
Age per year	1.02	1.00–1.04	0.046	1.02	0.99–1.05	0.153
Male sex	1.20	0.76–1.91	0.440			
BMI per kg/m^2^	0.92	0.87–0.97	0.002	0.90	0.84–0.97	0.004
CRT-D	1.11	0.62–1.99	0.725			
Year of CRT device implantation	0.90	0.83–0.96	0.003	1.01	0.91–1.12	0.895
Ischemic cardiomyopathy	1.77	1.20–2.62	0.004	0.98	0.58–1.65	0.928
Diabetes mellitus	1.11	0.68–1.81	0.678			
Hypertension	0.68	0.45–1.02	0.065			
LVEF per %	0.96	0.94–0.99	0.005	0.97	0.94–0.99	0.034
LVESV per mL	1.00	1.00–1.01	0.065			
Atrial fibrillation	1.23	0.84–1.81	0.295			
QRS area > 132 µVs	1.04	0.71–1.52	0.854			
QRS duration > 160 ms	0.63	0.38–1.07	0.086			
∆QRS area > 35 µVs	0.99	0.67–1.46	0.957			
∆QRS duration > 47 ms	1.21	0.82–1.78	0.340			

HR, hazard ratio. CI, confidence interval. LVAD, left ventricular assist device. Htx, heart transplantation. BMI, body mass index. CRT-D, cardiac resynchronization therapy defibrillator. LVEF, left ventricular ejection fraction. LVESV, left ventricular end systolic volume.

**Table 4:** Univariate and multivariate binary logistic regression analysis for LVESV improvement of 15% (*n* = 142).

	OR	95% CI	*p*-Value	OR	95% CI	*p*-Value
	Univariate Analysis			Multivariate Analysis		
Age per year	1.04	1.01–1.07	**0.020**	1.03	0.99–1.07	0.098
Male sex	1.23	0.60–2.54	0.574			
BMI per kg/m^2^	0.99	0.91–1.07	0.802			
Year of CRT device implantation	0.90	0.79–1.03	0.122			
Ischemic cardiomyopathy	0.77	0.41–1.46	0.429			
Diabetes mellitus	0.36	0.14–0.93	0.034	0.30	0.11–0.83	0.021
Hypertension	2.62	1.34–5.11	0.005	2.52	1.21–5.25	0.014
LVEF per %	1.00	0.97–1.03	0.892			
AF	1.57	0.82–3.01	0.173			
QRS area > 132 µVs	2.23	1.16–4.29	0.017	2.23	1.10–4.50	0.026
QRS duration > 160 ms	0.91	0.31–2.66	0.869			
∆QRS area > 35 µVs	1.52	0.80–2.88	0.203			
∆QRS duration > 47 ms	1.99	1.04–3.80	0.037	1.69	0.84–3.40	0.144

LVESV, left ventricular end systolic volume. OR, odds ratio. CI, confidence interval. BMI, body mass index. LVEF, left ventricular ejection fraction. AF, atrial fibrillation.

**Table 5:** Overview of previously described QRS area, morphology and duration.

Reference	Median QRS Area µVs [IQR]	QRS Morphology	QRS Duration (ms)	Number of Patients
Current study	132 [65]	RV paced	188 ± 27	303
Current study	90 [61]	Biventricular paced	154 [37]	296
Emerek et al. [5]	77	Non-LBBB *	Unknown	240
Emerek et al. [5]	101	LBBB	156 ± 21	465
De Pooter et al. [27]	145 [81]	LBBB	180 [30]	25
Marinko et al. [10]	74 [42]	Biventricular paced	152 ± 22.9	445
De Pooter et al. [27]	90 [60]	Biventricular paced	151 [24]	25

RV, right ventricular. LBBB = left bundle branch block. * Includes RV paced, right bundle branch block, and intraventricular conduction disorder.

## Data Availability

Data are available from the corresponding author upon reasonable request.

## Abbreviations

The following abbreviations are used in this manuscript:

ACE	Angiotensin-converting enzyme
BMI	Body mass index
CCMO	Centrale Commissie Mensgebonden Onderzoek
CI	Confidence interval
CRT	Cardiac resynchronization therapy
CRT-D	Cardiac resynchronization therapy defibrillator
ECG	Electrocardiogram
HFH	Heart failure hospitalization
HR	Hazard ratio
ICD	Implantable cardioverter defibrillator
LBBB	Left bundle branch block
LV	Left ventricular
LVAD	Left ventricular assist device
LVEF	Left ventricular ejection fraction
LVESV	Left ventricular end systolic volume
LVLW	Left ventricular lateral wall
Ms	Milliseconds
MUG	Maastricht–Utrecht–Groningen
NYHA	New York Heart Association
OR	Odds ratio
RV	Right ventricular
